# Nonlinear mixed effects modeling of gametocyte carriage in patients with uncomplicated malaria

**DOI:** 10.1186/1475-2875-9-60

**Published:** 2010-02-26

**Authors:** Greg B Distiller, Francesca Little, Karen I Barnes

**Affiliations:** 1 Department of Statistical Sciences, University of Cape Town, Cape Town, South Africa; 2 Department of Pharmacology, University of Cape Town, Cape Town, South Africa

## Abstract

**Background:**

Gametocytes are the sexual form of the malaria parasite and the main agents of transmission. While there are several factors that influence host infectivity, the density of gametocytes appears to be the best single measure that is related to the human host's infectivity to mosquitoes. Despite the obviously important role that gametocytes play in the transmission of malaria and spread of anti-malarial resistance, it is common to estimate gametocyte carriage indirectly based on asexual parasite measurements. The objective of this research was to directly model observed gametocyte densities over time, during the primary infection.

**Methods:**

Of 447 patients enrolled in sulphadoxine-pyrimethamine therapeutic efficacy studies in South Africa and Mozambique, a subset of 103 patients who had no gametocytes pre-treatment and who had at least three non-zero gametocyte densities over the 42-day follow up period were included in this analysis.

**Results:**

A variety of different functions were examined. A modified version of the critical exponential function was selected for the final model given its robustness across different datasets and its flexibility in assuming a variety of different shapes. Age, site, initial asexual parasite density (logged to the base 10), and an empirical patient category were the co-variates that were found to improve the model.

**Conclusions:**

A population nonlinear modeling approach seems promising and produced a flexible function whose estimates were stable across various different datasets. Surprisingly, dihydrofolate reductase and dihydropteroate synthetase mutation prevalence did not enter the model. This is probably related to a lack of power (quintuple mutations n = 12), and informative censoring; treatment failures were withdrawn from the study and given rescue treatment, usually prior to completion of follow up.

## Background

Gametocytes are the sexual form of the malaria parasite and the main agents of transmission. Malaria is diagnosed based on the presence of asexual parasites and most anti-malarials act on these stages with the aim of eliminating them. However, of vital importance to control (and eventually eliminate) malaria and prevent the spread of drug resistance is the prevention and/or curtailment of gametocytemia [1,2].

While transmission of the malaria parasite is dependent on various factors such as vector characteristics, host susceptibility and climatic conditions, it is host infectivity (the probability of a mosquito becoming infected after biting an infected person) that plays a crucial role in the onward spread of an individual infection [3-5]. There are various factors that influence human host infectivity to mosquitoes and the density of gametocytes appears to be the best single measure available [3].

Despite the obvious importance of gametocyte carriage for both the transmission of malaria and the spread of resistance, little research has been conducted modeling gametocyte densities, and by extension host infectivity, directly. Rather, models generally rely on the more frequently measured asexual parasite densities from which gametocyte carriage is estimated by either making a simple assumption (such as a constant gametocyte switching rate), or by using a more advanced model such as using lagged values based on asexual parasite densities [6,7].

Yet the relationship between gametocytes and asexual parasites is not straightforward. While one would expect a higher asexual parasite load to be associated with a higher sexual parasite load, there are also other factors that could be associated with low asexual densities such as immunity, treatment or a long duration of infection. As some of these factors would also increase gametocyte carriage, the relationship in question is often confounded in a clinical setting [2,8].

The primary objective of this research was therefore to model gametocytes directly to produce gametocyte density curves over time for various patient profiles. These curves could then be used to estimate gametocyte densities and clearance rates, hence leading to a more realistic understanding of infectivity in the population. Distributions of gametocytemia have interesting features that need to be addressed as part of any statistical analysis or modeling. These include:

(i) Repeated measures: Patients are seen on up to nine occasions during a 42-day period and gametocyte densities are measured on seven of these occasions.

(ii) Study design: Studies are designed to monitor asexual parasite density as an indicator of therapeutic efficacy. Gametocyte emergence lags asexual parasite emergence and hence the intervals for measurement of gametocytemia are not ideal.

(iii) Informative censoring for treatment failures: Patients are withdrawn and given rescue treatment if parasites do not clear or if they re-emerge, resulting in censoring of gametocyte distributions. In this way the failure to clear asexual parasites is a competing event to the measuring of gametocytes, with the result that the gametocyte distributions are not seen or are incomplete for patients who are resistant to the anti-malarial being studied and hence are withdrawn due to treatment failure.

One way of dealing with the bias due to informative censoring is, or could be, by imputation of the missing parts of the gametocyte distributions. To do this, a method that directly models the gametocyte density-time relationship for different groups of patients is needed.

## Methods

### Patient Data

Data for this research came from open label clinical trials of patients treated with sulphadoxine-pyrimethamine (SP) for uncomplicated symptomatic *falciparum *malaria at the Naas and Mangweni clinics (Mpumalanga province, South Africa) and Namaacha and Bela Vista Clinics (Maputo province, Mozambique). The study design focused on measuring the clinical and asexual parasitological response to treatment as recommended by the World Health Organization. Gametocyte densities were thus measured on days 0, 3, 7, 14, 21, 28, and 42 following treatment.

Of the 447 patients enrolled in these studies [9-11], a subset of 103 patients who had no gametocytes pre-treatment and who had at least three non-zero gametocyte density measurements (including 56 patients with at least four non-zero readings) were included in this analysis. Patients with gametocytes pre-treatment (n = 23) were excluded so that the analysis could focus on the development of gametocytes during the primary infection, rather than pre-existing gametocytes potentially from previous infections. Gametocyte density was counted on thick blood smears against 1,000 leukocytes assuming 8,000 leukocytes per microlitre (*μL*). This explains why the lowest detectable density was 8 per *μL *as the smallest possible count would have been one gametocyte per 1,000 leukocytes.

Patients who failed treatment (in that they failed to clear their asexual parasites) were withdrawn from the study to be given rescue treatment and effectively were lost to follow up. Due to the time lag between asexual parasites and sexual gametocytes, the distribution of gametocyte densities for these patients were often truncated or, in some cases, the patients were withdrawn prior to the emergence of gametocytes. This resulted in informative censoring of gametocyte density distributions for treatment failures.

The original gametocyte readings were logged to the base two for the modeling. It is common to log a measurement that is characterized by long right tails and where it is more meaningful to express increases in a multiplicative rather than additive manner. Using a logarithmic scale with base two allowed a one unit change in the response to be viewed as a doubling or halving. For example, the gametocyte elimination half-life is the time it takes for the density of gametocytes to halve.

The main problem with the data was the high prevalence of zero measurements and their associated uncertainty. Attempting to include many zeros in the data when trying to solve nonlinear mixed effect models led to estimation difficulties. Therefore there were two broad treatments of these zeros. In both cases the zero reading at day 0 was left at zero and patients exhibiting gametocytes on day 0 were excluded. In the first dataset (hereafter referred to as *Data 1*) all subsequent zeros were put to missing, while in the second instance (*Data 2*) they were changed to the detectable limit of eight gametocytes per microlitre (*μL*). Histograms were examined showing the overall frequency distributions and found that the shape of the distribution does not change qualitatively when we change the eight to a four or a twelve.

### Ethical considerations

These studies were approved by the Research and Ethics Committees of the University of Cape Town, the Mpumalanga Department of Health and the Mozambican Ministry of Health prior to commencement. These clinical studies were conducted in accordance with the South African Clinical Trials Guidelines [12] and the principles laid down by the World Health Assembly of 1975 on Ethics in Human Experimentation and the Helsinki Declaration. Written Informed consent was obtained from each subject or their guardian. In the case of illiteracy, an "X" mark was accepted as documentation of consent in the presence of an independent literate witness who also signed the consent form. Study subject identities were kept confidential. Those who chose not to participate in this study were treated with sulphadoxine-pyrimethamine but without routine follow up. At the time of the study, sulphadoxine-pyrimethamine and chloroquine monotherapies were the malaria treatment policies in Mpumalanga Province, South Africa and Mozambique, respectively.

### Empirical patient categories

**Figure 1:**
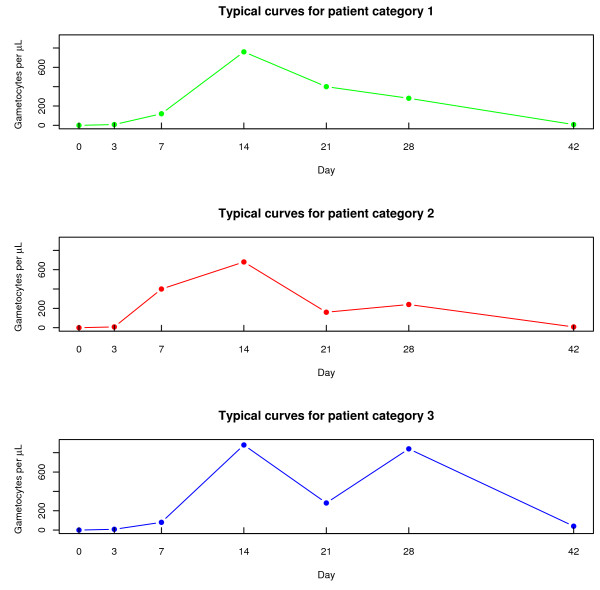
**Typical curves for the different patient categories**. Displays typical curves for the three empirically created patient categories.

Broadly speaking, three different patterns in the untransformed response were apparent across the sample of patients (see Figure 1). The grouping of patients was therefore an empirical exercise and the categories were created as follows:

group 1: this was the largest patient category and included patients that experienced an initial increase followed by a decrease that did not increase again.

group 2: the second group was for patients who experienced a second increase in gametocytes after the initial rise had started going down but the increase was slight i.e. less than a twofold increase.

group 3: the third category included those patients that experienced a marked second increase in gametocytes after the initial rise had started going down. The marked increase was defined as a change of at least twofold.

The three empirically created patient categories for the 103 patients included in the modeling were compared with regards to demographic and diagnostic variables in an attempt to see whether they could be explained by patient-specific features. Fisher's Exact test was used to assess dependence between the categorical variables due to small numbers in the cells, and the Kruskal-Wallis test was used to test for differences in continuous variables across groups given the skew distributions. The results are summarized in Table 1. The three empirically created patient groups did not clearly correspond to specific patient profiles as measured by the demographic and disease-specific co-variates. These groupings are most likely to reflect the effect of characteristics not documented in this study (e.g. delay in seeking treatment, degree of synchronicity, level of immunity).

**Table 1:** Univariate analysis of determinants of patient category.

Variable		Patient Category			Pvalue	Test
		1 (n = 74)	2 (n = 14)	3 (n = 15)		
Mutation	Sensitive	60 (73%)	9 (11%)	13 (16%)		1
	Resistant	7 (64%)	3 (27%)	1 (9%)	0.30	
Treatment	Success	66 (71%)	14 (15%)	13 (14%)		
Outcome	Failure	7 (78%)	0 (0%)	2 (22%)	0.47	
Site	Mpumulanga	50 (69%)	10 (14%)	12 (17%)		
	Maputo	24 (77%)	4 (13%)	3 (10%)	0.70	
Gender	Male	35 (70%)	7 (14%)	8 (16%)		
	Female	39 (74%)	7 (13%)	7 (13%)	0.95	

Parasite Clearance Time	Median	3	2	3	0.38	2
	IQ Range	2-3	2-7	3-7		
Age	Median	18	11	20		
	IQ Range	10-29	9-14	12-50	0.09	
Logged Parasite Density	Mean	4.5	4.5	4.6		
	Std Dev	0.5	0.5	0.5	0.37	

Summarises the results from exploring associations with patient category.

1 = Fisher's Exact test; 2 = Kruskal Wallis test.

The percentages shown are row percentages.

### Statistical Analysis

The gametocyte density-time profiles were typically nonlinear and were further characterized by both intra-individual variability due to repeated measures and inter-individual variability, which made nonlinear mixed effects modeling the appropriate statistical methodology. The modeling was conducted using R 2.2.0 [13] and in particular the *nlme *package [14].

Nonlinear mixed effects modeling is generally done in two stages:

1. Finding an underlying (nonlinear) function with a common structure that could be generating the data: *y*_*ij *_= *f *(*t*_*ij*_, ***β***_***i***_) + *e*_*ij *_where *y*_*ij *_is the observed response for the ith subject at the jth time point, *f *(*t*_*ij*_, ***β***_***i***_) refers to a function f whose structure is common to all patients with *t*_*ij *_representing time and parameters ***β***_***i ***_representing the value for the function's parameter(s) for the ith person, and *e*_*ij *_refers to the error or residual for the ith person at the jth time point and *e*_*ij *_~ *N *(0, *V*_*i*_) with *V*_*i *_being the intra-individual covariance matrix.

2. Inter-individual variability is modeled by adding random effects to mean parameters to allow individual specific responses, *β*_*i *_= *β *+ *b*_*i *_where *β*_*i *_is subject i's parameter value for the function and can be thought of as some random deviation (*b*_*i *_also know as a random effect) from the mean parameter value (*β*). It is assumed that *b*_*i *_~ *N *(0, *D*) where D is a (*k *× *k*) dispersion matrix for the k random effects, and that the *b*_*i *_are independent of each other and the *e*_*ij*_.

Co-variates can also be used to explain systematic variability in the actual parameter values between subjects: *β*_*i *_= *Aβ *+ *b*_*i *_where the matrix A is a design matrix formed from one or more co-variates.

With regards to the random effects, typically emphasis is placed not on the actual value of the random effects (*b*_*i*_'s) but rather the size of their variability. The covariance matrix of the random effects is specified with the model and includes both diagonal elements (variances of the random effects for particular parameters) and off-diagonal elements (covariance of the random effects between different parameters). More complicated structures are obviously more difficult to solve.

Various subsets of the data were modeled (not all shown here) and these models were compared by looking at both the Akaike Information Criteria's (AIC's), and at how robust the functions were across different datasets. The AIC is the appropriate statistic for comparing different models fitted by maximum likelihood. The aim was to find a function that fitted the data well, that made biological sense, and that was not overly sensitive to either the treatment of the zeros or the inclusion of different patient categories.

#### Finding the function

In total six different nonlinear functions were initially fitted to the different datasets. These functions included classic pharmacokinetic functions such as the one and two compartment models, the critical exponential (a variation of the double exponential), a reduced form of the critical exponential (hereafter called the "modified critical exponential"), and wave-like functions, such as the fourier and double fourier. Models based on different nonlinear functions were compared using the AIC statistic. Mixed effect modeling started with the most general and complex variance-covariance structure i.e. random effects for all function parameters were included and nonzero correlations amongst all of them were allowed. If this complex structure could be estimated, the results were examined for signs of possible improvement/simplification, i.e. non-significant and/or unnecessary random effects were dropped. If the model with the most general variance-covariance structure could not be estimated, a simpler structure was attempted. The resulting nested models were compared using likelihood ratio (LR) *χ*^2 ^statistics.

Use of the LR test in this strategy meant that Maximum Likelihood (ML) was used as opposed to Restricted Maximum Likelihood (REML) in the estimation process; any change to either the fixed or the random effects structure affected the REML likelihood and hence different structures could not be compared in this way if REML was used.

#### Adding co-variates

This phase examined if any co-variates could improve the model and explain some of the individual variation in the model parameters, thereby reducing the random effects. As suggested by Pinheiro and Bates [15], the strategy was to fit a model without any co-variates and then plot the random effects from this model against the potential co-variates. Any systematic pattern in the relationship between a potential co-variate and a particular random effect suggested that the particular co-variate be included in the model. Co-variates were added to the model one at a time starting with the one with the most obvious pattern. The new model was fitted and the process repeated with the new set of random effects. In addition to the LR test for nested models, statistical significance of any fixed effect associated with a co-variate was examined and various interactions were also explored.

The prospectively defined co-variates used in this analysis included: site (Mpumulanga vs Maputo province as the reference), age in years, gender (males versus females), day 0 parasite density (logged to the base 10), mutations associated with SP resistance (quintuple versus 0-4 mutations) [16], treatment outcome (failure versus success), parasite clearance time (either 1, 2, 3 or 7 days), and the empirical patient category described above.

## Results and Discussion

### Final function

Only the results from the two exponential functions as well as from the one-compartment model are presented along with AIC's for the two datasets (see Table 2). One of the salient features that emerged from the model-fitting was that the critical exponential function was clearly over-parameterized: in many of the models fitted neither the A nor the B parameter was significant. This was hardly surprising when considering the fact that Eastwood *et al *[17] used the critical exponential function to model gene expressions over time and referred to the A parameter as indicating the asymptotic response level, and the A+B parameter as determining the response at time = 0 (while the C and R parameters determined the shape of the curve). Since gametocyte density profiles needed to begin at zero and eventually clear i.e. end at zero, the A and B parameters should be set to zero thus resulting in the so called "modified critical exponential" function.

**Table 2:** Comparison of Models.

Function	Form*	AIC	
		Data 1	Data 2
One Compartment		1600	3008
Critical exponential	*A *+ (*B *+ *C *× *t*) × *R*^*t*^)	1772	2877
Modified critical exponential	(*C *× *t*) × (*R*^*t*^)	1869	2776

Compares the fit from the one compartment, critical exponential and the modified critical exponential functions.

* Note that t refers to time whereas *β*_*i*_, *A, B, C *and *R *refer to function parameters.

It was apparent that the AIC from the one compartment model was better than the other two functions for Data 1, but unfortunately the one compartment model did not perform well with Data 2. In Data 1 the zeros were dropped thereby allowing a smoother function to be fitted as the fitted curve did not need to reach down to the extremely small observations later on. When the zeros were replaced by a small number (in this case 8) in Data 2, the function then attempted to take these dips into account.

**Figure 2:**
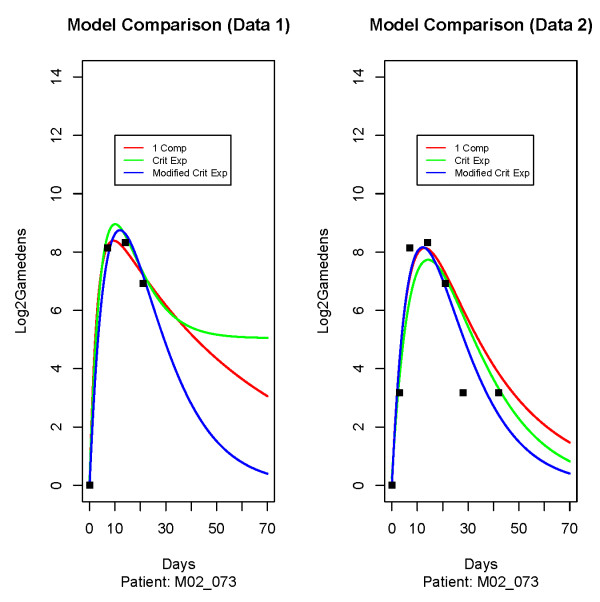
**Comparing different functions according to treatment of zeros**. Displays the fitted curves for a particular patient for the one compartment model, the critical exponential and the modified critical exponential function applied to both datasets.

Figure 2 shows the fitted curves for a particular patient for the one compartment model, the critical exponential and the modified critical exponential function applied to both datasets. The three points on the same horizontal band in the right hand panel refers to the three zeros for this patient that were ignored in the 1st dataset but were now assumed to be eight gametocytes *μL*. This figure clearly shows how using the one compartment model with Data 1 led to a much flatter right-hand tail that corresponded to a very slow elimination of gametocytes that did not appear to be biologically plausible. Therefore, the one compartment model could not have been chosen despite having lower AIC's for the models fitted to Data 1, and Data 2 was considered a preferable treatment of the zeros.

The results from fitting the two exponential functions to the two datasets are summarized in Table 3. Note that a random effect between two parameters refers to the correlation between those two random effects (e.g. *ρ*_*CR*_), whereas a random effect for a single parameter is expressed as a standard deviation (e.g. *σ*_*C*_).

**Table 3:** Critical exponential model & modified version.

Model		Fixed Effects (std error)				Random Effects			
		A	B	C	R	*σ*_*C*_	*σ*_*R*_	*ρ*_*CR*_	AIC
Critical Exponential	Data 1	5.04 (0.239)	-4.90 (0.274)	1.96 (0.059)	0.88 (0.005)	N/A	0.025	N/A	1772
	Data 2	0.04 (0.577)	0.19 (0.620)	1.54 (0.056)	0.93 (0.003)	0.33	N/A	N/A	2877

Modified version	Data 1	Fixed at 0	Fixed at 0	1.86 (0.072)	0.93 (0.002)	0.64	0.016	-0.79	1869
	Data 2	Fixed at 0	Fixed at 0	1.68 (0.064)	0.93 (0.002)	0.57	0.015	-0.77	2776

Summarises the results from fitting the two exponential functions.

For the critical exponential function fitted to Data 1, the estimated A and B parameters cancelled each other out and the lack of a zero or near to zero reading at the last time point resulted in a biologically implausible asymptotic level of approximately 5 (or 2^5 ^= 32 *μL*). For Data 2, both the A and B parameters were estimated to be essentially (or close to) zero.

The modified critical exponential function (where the A and B parameters were fixed to zero) was the final function chosen as most appropriate for this data. This decision was based on the clear stability that this structure demonstrated across various datasets (including a subset of patients that had at least four non-zero gametocyte density readings), particularly with regards to the parameter R. It is worth noting that the chosen function has only two fixed effect parameters and that the data did not seem able to support more complicated functions. For Data 2, fixing the A and B parameters to equal zero enabled us to estimate random effect components for the R parameter, and resulted in a better fitting model (AIC = 2776 vs 2877).

The modified critical exponential function is similar to an ordinary exponential decay curve . This is apparent when it is rewritten as *y *= (C * t) × *exp*^(*logRt*) ^where *logR *can be substituted for -*β*_1_, thereby constraining R to fall between zero and one in order to give a negative value. The difference is that the first term (C * t) is not constant like *β*_0 _but rather depends on time. The ordinary exponential decay curve has the property that the ratio of the response at successive time points () is equal across the range of the function. This property does not hold for the modified critical exponential function.

**Figure 3:**
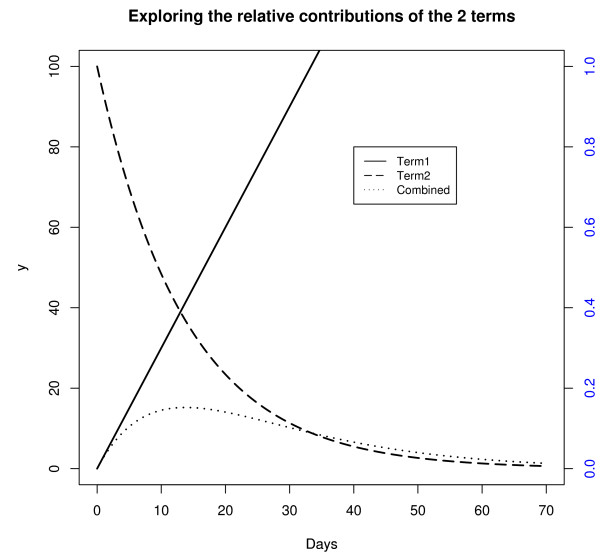
**Exploring the modified critical exponential curve**. Examines the relative contributions of the two terms in the modified critical exponential function.

The modified critical exponential function was explored further by examining the relative contributions of the two terms in the function (plotted in Figure 3). Note that the second term was plotted against the Y axis on the right that was on a different scale to the Y axis on the left. The first term (C * t) increased linearly with t. The contribution of the second term (*exp*^*logRt*^) was to exponentially decay the linearly increasing amount from the first term, with higher values for R leading to a slower rate of decay. The fact that term one increases with X is what differentiates this function from an ordinary exponential decay curve and allows the function to increase initially, provided C is estimated to be positive.

### Final model

The second stage of modeling resulted in the co-variates age, site (Mpumulanga vs Maputo province), logged baseline parasite density, and empirical patient category (pcat) being included in the model. The final model formulation was thus as follows:

The first stage (within-subject) of the model for individual *i *at time *j*:

and: *e*_*ij *_~ *N *(0, *V*_*i*_)

and the second stage of the model could be written as:

where site compared Mpumulanga province to Maputo province and pcat 2 and pcat 3 were two dummy variables for the patient category variable that used the first category as the reference category. Parameter estimates (along with 95% confidence intervals) are given in Table 4.

**Table 4:** The Final Model.

Parameters			Final Model	(95% CI)
**Fixed Effects**	**C**	**Intercept **(*β*_*C*0_)	2.1077	(1.8851 : 2.3303)
		**Mpm vs Map **(*β*_*C*1_)	-0.4988	(-0.7503 : -0.2472)
		**Pcat2 vs 1 **(*β*_*C*2_)	-0.0590	(-0.3810 : 0.2630)
		**Pcat3 vs 1 **(*β*_*C*3_)	-0.4910	(-0.7989 : -0.1832)
	
	**R**	**Intercept **(*β*_*R*0_)	0.8846	(0.8685 : 0.9007)
		**Mpm vs Map **(*β*_*R*1_)	0.0153	(0.0093 : 0.0213)
		**Age (years) **(*β*_*R*2_)	0.0002	(0.0001 : 0.0003)
		**log_10 _(pdens) **(*β*_*R*3_)	0.0061	(0.0027 : 0.0095)
		**Pcat2 vs 1 **(*β*_*R*4_)	0.0106	(0.0032 : 0.0179)
		**Pcat3 vs 1 **(*β*_*R*5_)	0.0134	(0.0058 : 0.0210)

**Random Effects**		*σ*_*C*_	0.4854	(0.3964 : 0.5944)
		*σ*_*R*_	0.0101	(0.0078 : 0.0130)
		*ρ*_*CR*_	-0.7447	(-0.8479 : -0.5872)

**Whole model**		**AIC**	2727.1	NA
		**Log Lk**	-1349.5	NA
		**Residual error**	1.3703	(1.2864 : 1.4597)

Summarises the results (with confidence intervals) from the final model.

**Figure 4:**
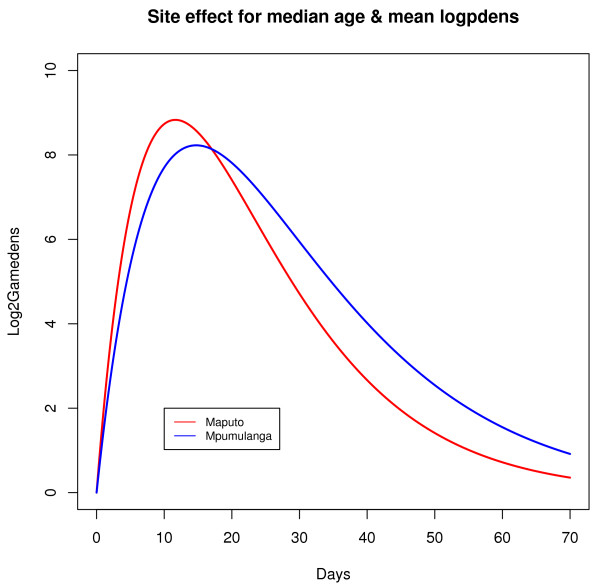
**Site effect**. Curves generated from the model that illustrate the site effect.

**Figure 5:**
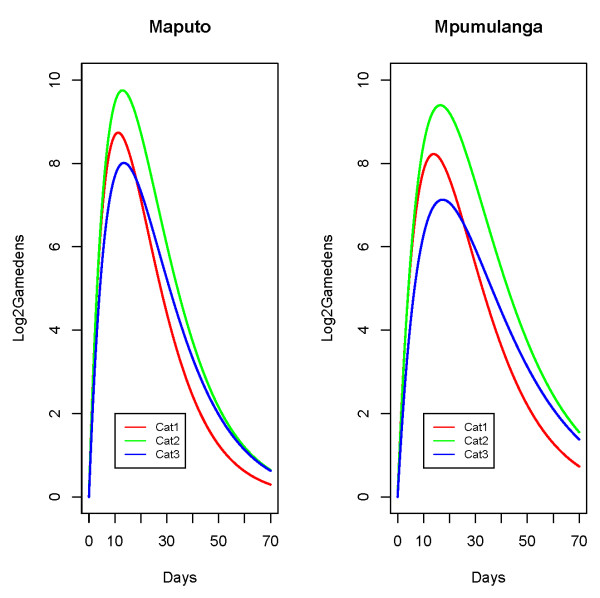
**Patient category effect**. Curves generated from the model that illustrate the patient category effect.

The effects of the co-variates were visually explored by examining curves generated from the final model and are summarized briefly here. The effects of the categorical variables are illustrated in Figure 4 and Figure 5 (for median age and baseline parasite density).

Patients from Mpumulanga tended to reach lower gametocyte densities compared to patients from Maputo and had slower rates of clearance (as indicated by a negative value for *β*_*C*1 _and a positive value for *β*_*R*1_, while older people and people with high pre-treatment asexual parasite densities tended to have higher gametocyte densities (Table 4 shows that the R parameter varied according to age and baseline parasite density level).

The site effect could reflect the level of immunity as malaria transmission in Maputo, Mozambique is much more intense (> 20-fold higher prevalence) than in Mpumulanga, South Africa - and delayed treatment seeking in partially immune patients may explain the faster increase as well as the faster clearance rate seen in Mozambique. The site effect could also reflect other unmeasured characteristics such as genetic differences.

Since 1900 it has been recognized that the frequency and density of asexual parasites decreases with increasing age in areas with stable malaria transmission as a result of partial immunity being acquired [6,18]. Unfortunately there is no direct measure of this immunity, so age is often used as a proxy to measure the effect of acquired immunity on various clinical and parasitological outcomes (asexual parasite density, failure rates, severe malaria rates) [6,19]. As immunity increases with age in areas of stable malaria transmission, lower immunity in children would result in children seeking treatment earlier than adults, explaining the slightly higher gametocyte densities in adults. As expected, higher asexual parasite densities at baseline were related to higher gametocyte densities.

Lastly, the model predicted that patients in category two, on average, had a higher and later peak than category one but with a very similar rate of increase and decrease. On the other hand, the model predicted that patients in the third category had a lower peak that occurred slightly later and then cleared more slowly, i.e. these patients would have carried gametocytes for longer. This made sense when one considered that patients in the third category had a second (and sometimes third) wave of gametocytes, and that the function used in the model coped with this by smoothing out these peaks, thereby resulting in a lower average peak and a slower clearance rate.

It was interesting that there was very little difference in the effect of the other co-variate parameters when excluding patient category. This suggested that the patient categories could be viewed as a proxy for characteristics that were not measured (ie immunity, duration of primary infection and degree of synchronicity), yet were also independent from the available co-variate information.

### Model fit

**Figure 6:**
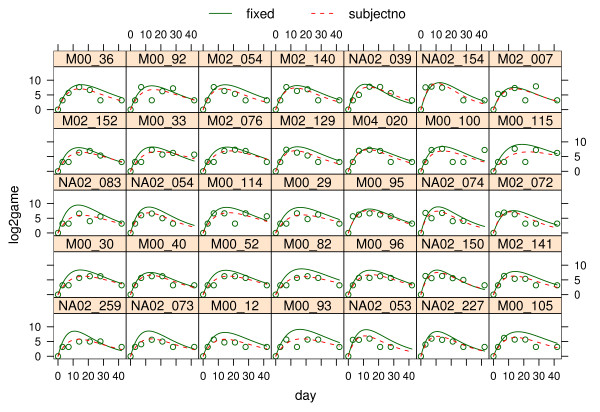
**Fitted vs observed data**. Displays fitted lines to observed profiles for a subset of patients. The fixed curves represent population curves based on the fixed effects whereas the curves displayed with broken lines (labelled "subjectno") represent curves for individual patients that are obtained by adding random effects to the fixed effects.

The adequacy and validity of the fitted models were assessed using an analysis of residuals that confirmed that all assumptions regarding normality and constant variance were met. Figure 6 compares fitted lines to observed profiles for a subset of patients. The chosen function appeared to be capable of taking on a wide array of shapes with just two parameters. These plots show that the population curves in general approximated the observed data fairly well and that adding random effects improved the fit for the different individuals.

## Conclusions

Host infectivity is a critical component in the transmission of malaria and the spread of anti-malarial drug resistance. While various factors are thought to influence host infectivity, the best indicator appears to be gametocyte density. The huge obstacle that drug resistance poses for the control of malaria together with the role that gametocytes play in the spread of such resistance makes it critical to examine gametocyte carriage more closely.

However, most malaria transmission models are based on asexual parasite distributions that infer gametocyte densities through assumed switching rates. Yet the relationship between asexual and sexual parasites is complex and so models that can cope with modeling gametocyte data directly are desirable. The model developed in this research aimed to make a contribution by modeling gametocytes directly. The study design from which this data was obtained (which is used for most clinical trials) used measurement points that were based around the expected clinical and asexual parasitological response to malaria treatment, and hence patients were seen fairly intensively during the first week of their infection and then less intensively later on, i.e. a gap of 14 days between the last two time points. Since the distribution of gametocytes lags that of asexual parasites the current design was not optimal for measuring gametocytes and consequently non-zero gametocyte densities were relatively sparse. This problem was exacerbated by the withdrawal of patients who failed treatment so that they could be given rescue treatment.

From a public health perspective, accurately describing gametocyte carriage (and hence infectivity) is of greater relevance at the population level than for individual patients and hence a population modeling approach was used to overcome the limitations of sparse data. The model developed with the modified critical exponential function seems able to encompass a wide range of variability in the curves, and appeared to be stable across the different datasets. The main use of the derived model would be to simulate curves that could be used to impute truncated/censored gametocyte distributions possibly using a multiple imputation method http://www.missingdata.org.uk.

The spread of resistant infections results from their increased gametocytaemia [16]. Thus it was surprising that the resistant (quintuple) mutation variables did not enter the model. This may be related to power as there were very few patients (n = 12) included with highly resistant infections. Including those patients with gametoytes pre-treatment (many of which would be recrudescent infections) would improve power, as these patients had a greater tendency to carry the quintuple mutation [8/23 (35%) vs 12/103 (12%), pvalue = 0.001], though as stated above the objective was to model gametocyte densities during the primary infection. Furthermore, most treatment failures were rescued and withdrawn from the study prior to completion of follow up. This informative censoring makes it very difficult to accurately estimate these gametocyte density-time profiles in resistant infections. Either a questionable assumption (such as equating their curves to those cured) has to be made, or a different study design that monitors gametocytes after patients have been withdrawn is needed. The issue of informative censoring in gametocyte data is an avenue that needs to be explored further. More data containing patients with resistant infections would increase the power to detect a "mutation effect" and hence allow the estimation of curves for treatment failures.

Lastly, this research was limited to carriers of gametocytes. In order to model the dynamics of malaria transmission and the spread of resistance in a population, the determinants of gametocyte prevalence should be assessed as reported previously using zero-inflated models used by Barnes *et al *[16,20].

## Competing interests

The authors declare that they have no competing interests.

## Authors' contributions

All authors had equal contribution in preparing this article. GD drafted the first manuscript of this article based on his MSc thesis, which was supervised by FL and KB. FL was integrally involved in the conceptualization of the analytic approach. KB was responsible for the design of the in vivo therapeutic efficacy studies which provided data for these analyses, and the biological interpretation of model results. All authors read and approved the final manuscript.

## References

[B1] Targett G Drakeley C Jawara M von Seidlein L Coleman R Deen J Pinder M Doherty T Sutherland C Walraven G Milligan P Artesunate reduces but does not prevent posttreatment transmission of Plasmodium falciparum to Anopheles gambiae J Infect Dis 2001 183 1254 1259 10.1086/319689 11262208

[B2] Barnes K White NJ Population biology and antimalarial resistance: The transmission of antimalarial drug resistance in Plasmodium falciparum Acta Trop 2005 94 230 240 15878154 10.1016/j.actatropica.2005.04.014

[B3] Draper CC Observations on the infectiousness of gametocytes in hyper-endemic malaria Trans R Soc Trop Med Hyg 1953 47 160 165 10.1016/0035-9203(53)90072-8 13077714

[B4] Diebner HH Eichner M Molineaux L Collins WE Jeffery GM Dietz K Modelling the transition of asexual blood stages of Plasmodium falciparum to gametocytes J Theor Biol 2000 202 113 127 10.1006/jtbi.1999.1041 10640432

[B5] Killeen G Ross A Smith T Infectiousness of malaria-endemic human populations to vectors Am J Trop Med Hyg 2006 75 38 45 16931814 10.4269/ajtmh.2006.75.2_suppl.0750038

[B6] Pongtavornpinyo W Mathematical modelling of antimalarial drug resistance PhD thesis 2006 School of Tropical Medicine, University of Liverpool

[B7] Ross A Killeen G Smith T Relationships between host infectivity to mosquitoes and asexual parasite density in *Plasmodium falciparum* Am J Trop Med Hyg 2006 75 2 Supplement 32 37 16931813 10.4269/ajtmh.2006.75.32

[B8] Price R Nosten F Simpson J Luxemburger C Phaipun L ter Kuile F Vugt MV Chongsuphajaisiddhi T White N Risk factors for Gametocyte carriage in uncomplicated Falciparum malaria Am J Trop Med Hyg 1999 60 1019 1023 10403336 10.4269/ajtmh.1999.60.1019

[B9] Barnes K Little F Smith P Evans A Watkins M White N Sulfadoxine-pyrimethamine pharmacokinetics in malaria: Pediatric dosing implications Clin Pharmacol Ther 2006 80 582 596 10.1016/j.clpt.2006.08.016 17178260

[B10] Mabuza A Govere J Durrheim D Mngomezulu N Bredenkamp B Barnes K Sharp B Therapeutic efficacy of sulfadoxine-pyrimethamine for Plasmodium falciparum malaria S Afr Med J 2005 95 346 9 15931450

[B11] Mabuza A Govere J Durrheim D Mngomezulu N Bredenkamp B Barnes K Sharp B Therapeutic efficacy of sulfadoxine-pyrimethamine in uncomplicated Plasmodium falciparum malaria 3 years after introduction in Mpumalanga S Afr Med J 2001 91 975 8 11847920

[B12] Guidelines for good practice in the conduct of clinical trails in South Africa Tech. rep., Department of Health, South Africa 2000

[B13] R Development Core Team R: A language and environment for statistical computing R Foundation for Statistical Computing, Vienna, Austria 2005 http://www.R-project.org

[B14] Pinheiro J Bates D DebRoy S Sarkar D nlme: Linear and nonlinear mixed effects models 2006 [R package version 3.1-68.1]

[B15] Pinheiro J Bates D Mixed-effects Models in S and S-PLUS 2002 Statistics and Computing, New York: Springer-Verlag

[B16] Barnes K Little F Mabuza A Mngomezulu N Govere J Durrheim D Roper C Watkins B White N Increased Gametocytemia after Treatment: An Early Parasitological Indicator of Emerging Sulfadoxine-Pyrimethamine Resistance in Falciparum Malaria J Infect Dis 2008 197 1605 1613 10.1086/587645 18471066

[B17] Eastwood DC Mead A Sergeant MJ Burton KS Statistical modelling of transcript profiles of differentially regulated genes BMC Mol Biol 2008 9 10.1186/1471-2199-9-66 18651954 PMC2525656

[B18] Baird JK Age-dependent characteristics of protection v. susceptibility to Plasmodium falciparum Ann Trop Med Parasitol 1998 92 367 390 10.1080/00034989859366 9683890

[B19] Aron JL May RM Anderson RM The poulation dynamics of Malaria Population Dynamics of Infectious Diseases, Population and Community Biology 1982 London: Chapman and Hall 139 179

[B20] Barnes K Durrheim D Little F Jackson A Mehta U Allen E Dlamini S Tsoka J Bredenkamp B Mthembu D White N Sharp B Effect of artemether-lumefantrine policy and improved vector control on malaria burden in KwaZulu-Natal, South Africa PLoS Med 2005 2 e330 10.1371/journal.pmed.0020330 16187798 PMC1240068

